# Investigation of the Effect of Type 2 Diabetes Mellitus on Subgingival Plaque Microbiota by High-Throughput 16S rDNA Pyrosequencing

**DOI:** 10.1371/journal.pone.0061516

**Published:** 2013-04-22

**Authors:** Mi Zhou, Ruichen Rong, Daniel Munro, Chunxia Zhu, Xiang Gao, Qi Zhang, Qunfeng Dong

**Affiliations:** 1 State Key Laboratory Breeding Base of Basic Science of Stomatology, Key Laboratory of Oral Biomedicine Ministry of Education, School and Hospital of Stomatology, Wuhan University, Wuhan, Hubei, China; 2 Department of Biological Sciences, University of North Texas, Denton, Texas, United States of America; 3 Department of Endodontics, School of Stomatology, Tongji University, Shanghai, China; 4 Department of Computer Science and Engineering, University of North Texas, Denton, Texas, United States of America; Queens University Belfast, Ireland

## Abstract

Diabetes mellitus is a major risk factor for chronic periodontitis. We investigated the effects of type 2 diabetes on the subgingival plaque bacterial composition by applying culture-independent 16S rDNA sequencing to periodontal bacteria isolated from four groups of volunteers: non-diabetic subjects without periodontitis, non-diabetic subjects with periodontitis, type 2 diabetic patients without periodontitis, and type 2 diabetic patients with periodontitis. A total of 71,373 high-quality sequences were produced from the V1-V3 region of 16S rDNA genes by 454 pyrosequencing. Those 16S rDNA sequences were classified into 16 phyla, 27 classes, 48 orders, 85 families, 126 genera, and 1141 species-level OTUs. Comparing periodontally healthy samples with periodontitis samples identified 20 health-associated and 15 periodontitis-associated OTUs. In the subjects with healthy periodontium, the abundances of three genera (*Prevotella*, *Pseudomonas*, and *Tannerella*) and nine OTUs were significantly different between diabetic patients and their non-diabetic counterparts. In the subjects carrying periodontitis, the abundances of three phyla (*Actinobacteria*, *Proteobacteria*, and *Bacteriodetes*), two genera (*Actinomyces* and *Aggregatibacter*), and six OTUs were also significantly different between diabetics and non-diabetics. Our results show that type 2 diabetes mellitus could alter the bacterial composition in the subgingival plaque.

## Introduction

Periodontitis is an inflammatory disorder, in which dental plaque bacteria induce exacerbated host immune response that destroys the gingival epithelium and alveolar bone, eventually leading to loss of teeth [1]. It is well established that the bacterial composition in periodontitis is different than that associated with healthy periodontium ([2], [3]). For example, periodontitis bacterial communities are more diverse than those in healthy tissue [4], and the disease microbiome harbors significantly enriched metabolic pathways adapted for oxygen-poor environments as well as lipid degradation pathways associated with known virulence-related activities [5].

Chronic periodontitis is often associated with diabetic patients with poor glycemic control ([6], [7], [8], [9], [10]). Diabetes mellitus significantly contributes to the severity, prevalence, and progression of periodontal disease ([11], [12], [13], [14]). Elevated oxidative stress responses, inflammatory cytokines, and receptor for advanced glycation end products (RAGE)-mediated damages have been observed under diabetic conditions in response to periodontal pathogens ([15], [16], [17], [18], [19], [20], [21] [22], [23], [24]), illustrating that diabetes mellitus significantly increases the host hyper-inflammatory response to periodontitis [14].

Besides affecting host immune response, diabetes mellitus also changes the oral environment, which may result in a different periodontal bacteria community than that in non-diabetic conditions. For example, increased gingival crevicular fluid glucose levels in diabetic patients [25] could provide an altered source of nutrition affecting the growth of certain bacterial species [26]. However, the extent of such effects on the subgingival microbiota composition still remains unclear ([14], [26]). Previous studies were mainly based on traditional methods such as checkerboard DNA-DNA hybridization and PCR to investigate the differences of the selected subgingival bacteria in diabetics compared with non-diabetics (*e.g.*, [27], [28], [29], [30], [31], [32], [33], [34], [35]). Instead of surveying the entire bacterial community, such traditional methods mainly suffered from limited detection of a small number of selected species [3]. For example, Field *et al.* [35] recently compared subgingival plaque microbiota in different backgrounds of periodontitis and diabetes mellitus, but their study was limited by the scope of quantitative PCR. They were only able to evaluate three bacterial species (*Aggregatibacter actinomycetemcomitans*, *Fusobacterium nucleatum*, and *Porphyromonas gingivalis*) in subgingival plaque and found no significant differences of these bacteria between type 2 diabetes mellitus patients and non-diabetic controls.

The aim of the present study was to examine whether diabetes mellitus might affect subgingival bacterial composition by high-throughput 16S rDNA sequencing with the 454 pyrosequencing technology [36], which has been widely adopted by numerous human microbiome projects including several studies characterizing the oral microbiome (e.g., [37], [38], [39], [40], [41], [42], [43]). Our study design included non-diabetic subjects without periodontitis (P−D−), non-diabetic subjects with periodontitis (P+D−), type 2 diabetic patients without periodontitis (P−D+), and type 2 diabetic patients with periodontitis (P+D+). The comparisons of the P−D− vs. P+D− and P−D+ vs. P+D+ groups were critical for discovering health-associated and periodontitis-associated bacteria in both diabetes-negative and diabetes-positive backgrounds, which were essential for understanding the interactions between periodontitis and diabetes mellitus. Based on our knowledge, our study is the first to apply 454 pyrosequencing to the above four groups of subjects.

While this manuscript was being prepared, Casarin *et al.* [44] reported a survey on the subgingival biodiversity in type 2 diabetic subjects by 16S rDNA sequencing using the traditional cloning-based Sanger sequencing method. Three important differences exist between our study and that of Casarin *et al.* [44]. First, Casarin *et al.* only included P+D+ and P+D− in their study. Second, Casarin *et al.* used paper points to collect subgingival biofilm, whereas we used a curette as the sample collection instrument. Paper points are generally limited to sampling the flowing or loosely adherent plaque, while a curette can also collect the more tightly-attached tooth or epithelium plaque ([45], [46]). Third, we were able to achieve much deeper sequencing depth with 454 pyrosequencing than that of the traditional Sanger sequencing used by Casarin *et al.* Overall, our study presents a distinct examination of the effects of diabetes mellitus on the subgingival bacterial community.

## Materials and Methods

### Ethics statement

Human subjects participated in the study after they signed the written informed consent in accordance with the study protocol approved by the Ethics Committee of the Faculty of Medicine for Human Studies, School & Hospital of Stomatology, Wuhan University (protocol number 2011029).

### Sample collection

Thirty-one Chinese subjects satisfied the inclusion criteria, which required no usage of antibiotics or non-steroidal anti-inflammatory drugs and no smoking in the three months prior to the sampling. Those selected participants, between 30 and 65 years old, had at least 20 teeth without any clinical signs of oral mucosal disease or root caries. None of them were either pregnant or HIV positive. They had also not been previously treated with any periodontal therapy or surgery. Table S1 shows all the clinical parameters of the 31 participating subjects. Fisher's exact test (p-value = 0.88) and ANOVA test (p-value = 0.11) confirmed that no gender or age bias, respectively, existed among the four subject groups in this study. The sampling procedure was similar to what was used in Paster *et al.* [47], as briefly described below. Subjects with type 2 diabetes had been diagnosed for at least one year with HbA1c ≥6.5%, fasting plasma glucose test ≥7.0 mmol/L, or OGTT 2 hour glucose test ≥11.1 mmol/L. Periodontitis was defined by the following criteria: at least 30% of sites with probing depth and attachment loss, and more than four with probing depth ≥4 mm and clinical attachment loss ≥2 mm. Subgingival plaque samples were extracted from the four deepest sites of the molars in the participants using sterile Gracey curettes and transferred into 200 µL of phosphate-buffered saline (PBS) buffer for immediate freezing at −70°C.

### DNA extraction and sequencing

Total DNA was isolated with a Qiagen DNA MiniAmp kit (Qiagen, Valencia, CA, USA) following the manufacturer's instruction on the tissue protocol. The universal primers targeting the 16S rDNA V1–V3 hypervariable region were used for PCR amplification: forward primer (8F, 5′-AGAGTTTGATCCTGGCTCAG-3′) and reverse primer (533R, 5′-TTACCGCGGCTGCTGGCAC-3′). The V1–V3 region was chosen because it could provide good detection of the oral microbiome [48]. The PCR primers were also tagged by unique barcodes for multiplex sequencing. PCR amplification was performed in the 20 µL reactions with 2.5 mM dNTPs, 5 µM forward and reverse primers, 20–50 ng template DNA, 1× polymerase buffer, and Platinum Taq DNA Polymerase High-Fidelity enzyme 0.4 U (Life Technologies, USA). After initial denaturation at 95°C for 4 minutes, 25 cycles of PCR were performed (denaturation at 95°C for 30 s, annealing at 55°C for 30 s, and extension at 72°C for 30 s). PCR amplicons were purified using AxyPrep DNA Gel Extraction kit (Axygen Biosciences, USA) according to the manufacturer's protocol,and visualized by electrophoresis in 1% agarose gels. Purified DNA samples were diluted in 30 uL 1× TE; an equal volume 2× PicoGreen working solution was added for a total reaction volume of 60 µL in a minicell cuvette. Fluorescence was measured on a Turner Biosystems TBS-380 Fluorometer using the 465–485/515–575-nm excitation/emission filter pair. Following quantification, purified amplicons were combined in equimolar ratios into a single tube. After preparing amplicons using the emPCR Kit II (according to the manufacturer's protocol), pyrosequencing was carried out on a 454 Life Sciences Genome Sequencer FLX Titanium instrument (Roche, USA). All of the sequences and associated metadata were deposited to the NCBI Sequence Read Archive [49] under the accession number SRA062091.

### Sequence analysis

The Mothur software (version 1.23.0, [50]) was applied to process the sequence reads as previously described [51]. Briefly, sequence reads were deconvoluted into individual samples based on perfect match to the barcode sequences. Primers and barcodes were trimmed from each read and the trimmed sequences shorter than 200 bp were discarded. Low-quality and chimeric sequences were removed with default Mothur parameters. The remaining high-quality sequences were binned into species-level operational taxonomic units (OTUs), which is commonly defined by the level of 16S rDNA sequence similarity (i.e., ≥97% for a ‘species’-level phylotype, [52]) based on the average neighbor algorithm in the Mothur package. As reported previously, the resulting number of OTUs may represent an inflated number of true species ([53], [54]). Therefore, during our manual process of species-level classification, we also merged some OTUs of interest that matched to the same reference database sequences with high confidence (e.g., ≥97% identity), which is similar to other published species-level classification methods (e.g., [55]). To avoid bias caused by the different sequencing depths of samples, we followed the normalization procedure previously published in Hawlena *et al* [51]. Briefly, 1000 sequences were randomly selected without replacement from each sample. This step of random sampling was repeated 1000 times and averaged to get the mean size of each OTU per normalized sample for statistical comparison. Taxonomic classification (from phylum to genus level) of the sequence reads was performed by the RDP Classifier (version 2.4, [56]) with the default 0.8 confidence threshold. Classification of the selected OTUs to the species level was achieved by BLASTing the OTU sequences against the NCBI 16S rDNA collection [57], SILVA database version 114 [58], and HOMD database version 12 [59] with manual inspection of the alignment results (minimum percentage of identity and coverage in the alignments as 97% and 95%, respectively). UniFrac analysis and non-metric multidimensional scaling (NMDS) were performed using the R packages *phyloseq* [60] and *vegan* [61], respectively. Both Wilcoxon rank-sum test and Fisher's exact test were performed with customized scripts that were implemented with the freely available R software environment (http://www.r-project.org). Relative abundance of each OTU was used in the statistical tests. In order to exclude rare OTUs that might be associated with large random sampling errors, only the OTUs with at least 0.5% relative abundance in one or more of the sample groups (*i.e.*, P−D−, P−D+, P+D−, and P+D+) were tested for their differential abundance or prevalence by the Wilcoxon rank-sum test and Fisher's exact test, respectively.

## Results and Discussion

### Taxonomic classification of 16S rDNA sequences

From 31 participating subjects, 71,393 high-quality 16S rDNA sequences were obtained after filtering out low-quality, chimeric, and non-bacterial sequences. The sequencing depth was similar among different sample groups although a slightly smaller amount of sequences were generated from the P+D− group (2504±357 in P−D+, 2611±216 in P−D−, 2361±470 in P+D+, and 1662±323 in P+D−). Compared to the 16S study by Casarin *et al.* [44] in which 87.4 sequences per subject were generated using Sanger sequencing, we achieved a much deeper sequencing depth with the 454 pyrosequencing technology (*i.e.*, 2,302 sequences per subject on average). Of those high-quality sequences, 87.22% could be classified into 126 genera, which belong to 16 phyla, 27 classes, 48 orders, and 85 families. The average percentage of classified sequences at the genus level is 91.01%±3.07% for P−D+, 91.68%±5.47% for P−D−, 87.32%±7.31% for P+D+, and 87.52%±7.72% for P+D−. No obvious bias in the proportion of unclassifiable sequences among different sample groups was observed (p = 0.40 based on the ANOVA test). All the sequences were clustered into 1,141 species-level OTUs based on their shared sequence similarity.

### Effects of periodontitis on subgingival plaque microbiota compositions

**Figure 1:**
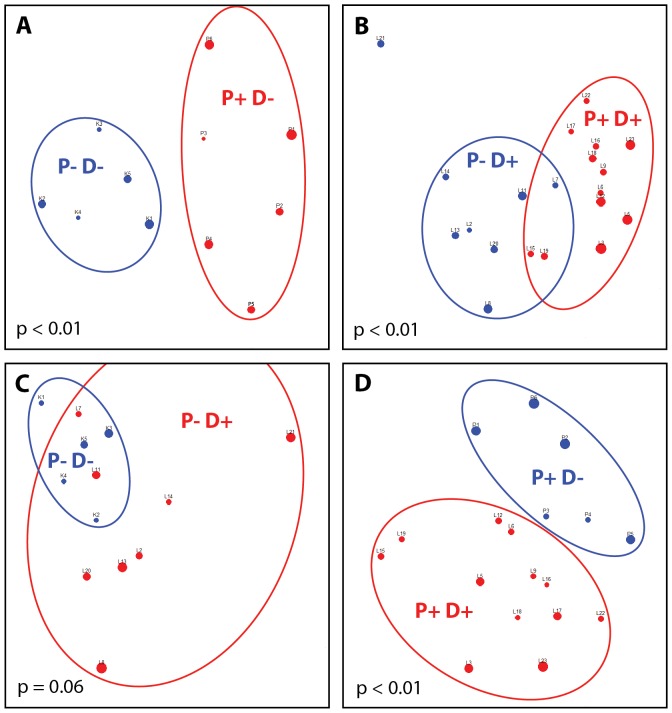
Subgingival plaque bacterial community composition comparison. The figure shows the results of non-metric multidimensional scaling (NMDS) applied to the unweighted UniFrac distances between different subsets of samples. The X- and Y-axes represent the first and second NMDS dimensions, respectively. The label next to each data point indicates the sample name. (A) Among diabetes-negative samples, a PERMANOVA test indicates significant (p<0.01) differences in the UniFrac distances according to the presence or absence of periodontitis. (B) A similar comparison among diabetes-positive samples is also significant (p<0.01). (C) Among periodontitis-negative samples, there is no clear separation based on diabetes status (p = 0.06). (D) In periodontitis-positive samples, however, significant differences do exist based on diabetes status (p<0.01).

The bacterial compositions at the community level in the different periodontitis groups were compared by unweighted UniFrac distance, which is commonly used in microbial ecology for quantitatively measuring compositional differences among microbial communities by calculating the length proportion of unique branches to each sample-specific taxon in a phylogenetic tree constructed from 16S rDNA sequences [62]. Figure 1 shows that the samples in the periodontitis-positive groups (*i.e.*, P+D− or P+D+) are well separated from those in the periodontitis-negative groups (*i.e.*, P−D− or P−D+) based on the unweighted UniFrac distances measured at the OTU level (Figures 1A and 1B), reflecting that the bacterial compositions in periodontitis and healthy samples were distinct. The significantly different bacterial communities between the periodontitis-negative and periodontitis-positive groups could also be confirmed by PERMANOVA test (p<0.01). Similar to our results, several other high-throughput 16S rDNA surveys also showed different subgingival bacterial compositions in non-diabetic subjects with periodontitis and healthy controls ([4], [5], [47], [63], [64]). Additionally, our results further show that compositional shifts in the subgingival plaque bacterial community associated with periodontitis also exist in diabetic patients.

Standard statistical methods (*i.e.*, Wilcoxon rank-sum test for relative abundance and Fisher's exact test for prevalence) were applied to pinpoint signature bacteria that were significantly different between the periodontitis-positive and periodontitis-negative samples. If the prevalence or relative abundances of the signature OTUs were significantly higher in the periodontitis-positive groups, the bacteria were designated as periodontitis-associated. Otherwise, the bacteria were designated as health-associated if they were significantly more abundant in periodontitis-negative groups. In total, 20 and 15 signature OTUs were designated as health-associated and periodontitis-associated bacteria, respectively (Tables 1 and S2). Many of our designations were consistent with previous studies ([47], [63], [64],[65], [66], [67], [68], [69], [70], [71], [72]), such as periodontitis-associated OTU0001 (*Porphyromonas gingivalis*), OTU0004 (genus of *Leptotrichia*), OTU0010 (*Tannerella forsythia*), OTU0011 (*Treponema denticola*), OTU2003 (*Treponema medium*), and OTU2006 (*Prevotella intermedia*), as well as heath-associated OTU0003 (*Corynebacterium matruchotii*), OTU0019 (*Neisseria elongate*), OTU0052 (*Streptococcus infantis*), and OTU0161 (*Capnocytophaga sputigena*) ([47], [63], [69], [70], [71], [73]). The reported roles of some other bacteria in periodontitis are often unclear. In addition, 13 signature OTUs were not classifiable at species or higher taxonomic levels (Table 1); therefore we were unable to validate their designated associations based on literature search. Such unclassified species might represent novel bacteria previously unknown to be involved in periodontitis.

**Table 1:** Health-associated and periodontitis-associated OTUs.

	Health-associated bacteria OTUs	Periodontitis-associated bacteria OTUs
Significance uniquely identified in the diabetes-negative background	OTU0003 *Corynebacterium matruchotii* OTU0016 family of *Propionibacteriaceae* OTU0020 *Prevotella loescheii* OTU0025 genus of *Aggregatibacter* OTU0026 *Selenomonas noxia* OTU0088 *Cardiobacterium hominis* OTU0094 *Neisseria flavescens* OTU0096 *Cardiobacterium valvarum* OTU0102 family of *Leptotrichiaceae* OTU0127 genus of *Leptotrichia* OTU0161 *Capnocytophaga sputigena* OTU2004 *Porphyromonas sp.*	OTU2001 *Selenomonas sputigena* OTU0507 family of *Prevotellaceae*
Significance identified in the both diabetes-positive and diabetes-negative backgrounds	OTU0052 *Streptococcus infantis* OTU2002 *Streptococcus gordonii* OTU2005 *Actinomyces naeslundii*	OTU0011 *Treponema denticola* OTU2006 *Prevotella intermedia* OTU0343 family of *Prevotellaceae*
Significance uniquely identified in the diabetes-positive background	OTU0019 *Neisseria elongate* OTU0028 *Rothia dentocariosa* OTU0194 genus of *Veillonella* OTU0214 *Haemophilus parainfluenzae* OTU0280 genus of *Neisseria*	OTU0001 *Porphyromonas gingivalis* OTU0004 genus of *Leptotrichia* OTU2003 *Treponema medium* OTU0009 order of *Bacteroidales* OTU0010 *Tannerella forsythia* OTU0017 family of *Synergistaceae* OTU0044 *Porphyromonas endodontalis* OTU0056 unclassified OTU0058 *Filifactor alocis* OTU0101 genus of *Leptotrichia*

All OTUs listed had significantly different abundances or prevalence between the healthy and periodontitis samples within the diabetes-negative group, diabetes-positive group, or in both groups, as indicated by the three rows. The two columns indicate whether each OTU was more abundant or prevalent in periodontitis-negative or periodontitis-positive samples.

From the diabetes-negative group, 20 species-level OTUs were identified as signature bacteria differentiating the P+D− from the P−D− samples. Similarly from the diabetes-positive group, 21 OTUs were identified as signature bacteria differentiating the P+D+ from the P−D+ samples (Tables 1 and S2). Out of all the signature OTUs, only six OTUs were shared in the above diabetes-positive and diabetes-negative groups, 14 OTUs were unique to the diabetes-negative group, and 15 OTUs were unique to the diabetes-positive group. The six shared OTUs were OTU0011 (*Treponema denticola*), OTU0052 (*Streptococcus infantis*), OTU0343 (family of *Prevotellaceae*), OTU2002 (*Streptococcus gordonii*), OTU2005 (*Actinomyces naeslundii*), and OTU2006 (*Prevotella intermedia*). Except for OTU0343, all the shared OTUs were reported previously as being associated with either periodontitis or healthy periodontium ([5], [47], [63], [64], [65], [66], [67]). For example, *T. denticola* is part of the “red complex” associated with periodontitis ([64], [67]). OTU0343 could only be classified as *Prevotellaceae* at the family level with high confidence. This OTU was designated as periodontitis-associated bacteria since its relative abundance was 3.15% in P+D− compared to 0.069% in P−D− samples (p-value = 0.028), and 0.99% in P+D+ compared to 0% in P−D+ samples (p-value = 0.025). The six shared OTUs may represent the core periodontal bacterial community that is commonly involved in pathogenesis or prevention of periodontitis regardless of diabetes status. The other 29 OTUs were identified as health- or periodontitis-associated bacteria only in either the diabetes-negative or the diabetes-positive group based on their small p-values in the statistical tests. However, 24 of those 29 OTUs displayed consistently higher (or lower) prevalence or abundance in periodontitis-negative samples compared to periodontitis-positive samples in both diabetes-positive and diabetes-negative groups, despite non-significant p-values in one of the groups. If larger sample sizes were obtained, statistically significant p-values could have potentially been achieved for those “unique” OTUs, leading to more observations of “shared” signature OTUs. For example, OTU2003 (*Treponema medium*) was only identified as periodontitis-associated in the diabetes-positive background. In the diabetes-negative background, the p-value of this species was not significant in the two-tailed Wilcoxon rank-sum test, but its relative abundance was clearly higher in the P+D− samples (3.21%) than in the P−D− samples (0.92%), indicating its association with periodontitis (one-tailed test does show significance, p = 0.04). On the contrary, five of those 29 signature OTUs did not follow the same trend, prompting the possibility that “health-associated” or “periodontitis-associated” bacteria might need to be defined in the context of diabetes. For example, the relative abundance of OTU0020 (*Prevotella loescheii*) in P+D− and P−D− samples was 0.33% and 5.05%, respectively, highly enriched in the healthy periodontium samples in the diabetes-negative background (p = 0.004). However, its relative abundance in P+D+ and P−D+ samples was 1.16% and 0.33%, respectively, displaying a trend of enrichment in the periodontitis samples in the diabetes-positive background.

### Effects of diabetes mellitus on subgingival plaque microbiota compositions

We also compared P−D− with P−D+ as well as P+D− with P+D+ to investigate the effects of diabetes mellitus on the subgingival plaque microbiota in subjects with or without periodontitis. Although the P−D− samples could not be easily separated from the P−D+ samples based on unweighted UniFrac distances (Figure 1C; PERMANOVA test, p = 0.06), three genera and nine OTUs had significantly different abundance between the P−D− and P−D+ samples (Tables 2 and S2). At the genus level, *Prevotella* (p = 0.019) and *Tannerella* (p = 0.042) were enriched in the P−D− samples while *Pseudomonas* (p = 0.045) was more associated with the P−D+ samples. At the OTU level, we were not able to designate OTU0125 (the order of *Actinomycetales*, unclassifiable at family level or below) or OTU0193 (the genus of *Prevotella*, unclassifiable at species level) as either health-associated or periodontitis-associated bacteria. All of the remaining seven OTUs could be considered as putative health-associated bacteria based on the above comparisons of their relative abundances in periodontitis-positive and periodontitis-negative groups. Only one of those seven health-associated OTUs, OTU0280 (genus of *Neisseria*), was enriched in the P−D+ samples. The other six health-associated OTUs significantly decreased their abundances in the P−D+ samples (p<0.05): OTU0003 (*Corynebacterium matruchotii*), OTU0020 (*Prevotella loescheii*), OTU0094 (*Neisseria flavescens*), OTU0096 (*Cardiobacterium valvarum*), OTU0102 (family of *Leptotrichiaceae*, unclassifiable at the genus level or below), and OTU0187 (*Capnocytophaga ochracea*; although the relative abundance of this OTU was not significantly different in the comparison of the periodontitis-negative and the periodontitis-positive groups, its association with healthy periodontitium has been reported in previous studies (*e.g.*, [73])). The tendency of reduced abundance of health-associated bacteria in the P−D+ samples might predispose the diabetic patients to greater risk of periodontitis.

**Table 2:** Signature OTUs associated with diabetic and non-diabetic samples.

		Health-associated OTUs	Periodontitis-associated OTUs	Other OTUs
Periodontitis negative	↑ in Diabetes	OTU0280 genus of *Neisseria*		OTU0125 order of *Actinomycetales*
	↓ in Diabetes	OTU0003 *Corynebacterium matruchotii* OTU0020 *Prevotella loescheii* OTU0096 *Cardiobacterium valvarum* OTU0094 *Neisseria flavescens* OTU0102 family of *Leptotrichiaceae* OTU0187 *Capnocytophaga ochracea*		OTU0193 genus of *Prevotella*
Periodontitis positive	↑ in Diabetes	OTU0016 family of *Propionibacteriaceae* OTU0161 *Capnocytophaga sputigena*	OTU0010 *Tannerella forsythia*	OTU0015 order of *Burkholderiales*
	↓ in Diabetes		OTU0343 family of *Prevotellaceae*	OTU0046 *Prevotella tannerae*

Within the periodontitis-negative and periodontitis-positive sample groups, the listed OTUs were significantly either more or less abundant in diabetes-positive samples than in diabetes-negative samples, as indicated by the rows. The three columns indicate whether each OTU was significantly enriched in periodontitis-negative samples, periodontitis-positive samples, or neither, as found in the comparisons shown in Table 1.

In the periodontitis-positive group, the P+D+ samples could be separated from the P+D− samples based on unweighted UniFrac distances (Figure 1D; PERMANOVA test, p<0.01). At the phylum level, both *Actinobacteria* (p = 0.0013) and *Proteobacteria* (p = 0.041) had significantly higher abundance in P+D+, while *Bacteroidetes* was more abundant in P+D− (p = 0.018). Casarin *et al.* [44] also detected the same trend for the above three phyla in diabetic subjects. At the genus level, *Actinomyces* (p = 0.0057) and *Aggregatibacter* (p = 0.00037) were more abundant or prevalent in the P+D+ samples. Both *Actinomyces* and *Aggregatibacter* were also observed by Casarin *et al.* [44] to be more associated with their P+D+ subjects. At the OTU level, six significantly different OTUs were detected between the P+D+ and P+D− samples in our study (Tables 2 and S2): OTU0015 (classified as *Burkholderiales* at the order level), OTU0046 (*P. tannerae*), OTU0016 (classified as *Propionibacteriaceae* at the family level), OTU0161 (*Capnocytophaga sputigena*), OTU0010 (*T. forsythia*) and OTU0343 (classified as *Prevotellaceae* at the family level). OTU0161 (*C. sputigena*) was designated as putative health-associated bacteria in our data set, which is also consistent with its reported association with healthy periodontal sites ([69], [70]). We observed that this species was more abundant in the P+D+ than in the P+D− samples. The increase of *Capnocytophaga* species in P+D+ subjects was also reported by Casarin *et al.* [44]. *C. sputigena* is a known glucose-fermenting species ([74], [73], [70]), which might explain its higher abundance in diabetic patients. Besides the species of *C. sputigena*, the relative abundance of OTU0016 (classified as *Propionibacteriaceae* at the family level) was also higher in the P+D+ samples. OTU0016 was designated as health-associated bacteria based on the above comparisons of its relative abundances in periodontitis-positive and periodontitis-negative groups, although it is difficult to verify the role of the unclassified species of *Propionibacteriaceae* in the literature. The physiological impacts of the higher abundance of such health-associated subgingival bacteria on periodontitis in diabetic patients are unclear.

In our data set, OTU0010 (*Tannerella forsythia*) was more likely to be detected in the P+D+ samples than in the P+D− samples. This OTU was detected in all 12 samples in the P+D+ group, while it was present in only three out of six total P+D− samples (Fisher's exact test, p = 0.025). Contradictory to our observations, the PCR results by Sardi *et al.* [34] and Campus *et al.* [75] (both using a curette) and 16S rDNA sequencing by Casarin *et al.* (using paper points) [44] indicated that *T. forsythia* is more prevalent in P+D− subjects. On the other hand, Li *et al.* (using paper points) [76] also detected higher prevalence and abundance of *T. forsythia* by PCR in P+D+ subjects, which is in agreement with our results. *T. forsythia* is a major component of the “red complex” associated with periodontitis ([64], [67]). Our result indicates that the higher prevalence of this well-known periopathogen in diabetic patients could contribute to their severity of periodontitis.

It is noteworthy to mention that we did not detect any significant difference for the other two components of the “red complex”, *P. gingivalis* and *T. denticola*, when comparing the P+D+ samples with the P+D− samples, although both species were identified as periodontitis-associated bacteria in our data set. *P. gingivalis* was the most abundant species in our periodontitis-positive samples. Its average relative abundance in the P+D− and P+D+ samples was 17.85% and 12.48%, respectively (p>0.05 in both Wilcoxon rank-sum test and Fisher's exact test). Our observation of *P. gingivalis* is supported by Yuan *et al.* [33], Sardi *et al.* [34], Field *et al.* [35], and Li *et al.* [76] (all of their results were based on PCR), although Campus *et al.* [75] and Casarin *et al.* [44] reported higher prevalence or abundance of this species in their P+D− subjects. The average relative abundance of *T. denticola* in our P+D− and P+D+ samples was 2.41% and 2.38%, respectively. Consistent with our results, Hintao *et al.* [30] (based on checkerboard DNA-DNA hybridization) and Yuan *et al.* [33] (based on PCR) also did not detect any significant difference for this species between their P+D− and P+D+ subgingival samples. In addition, we noticed that Casarin *et al.* [44] (based on 16S rDNA Sanger sequencing) did not find *T. denticola* to be significantly different in their study either, although Li *et al.* [76] observed a higher abundance and prevalence of this species in their P+D+ subjects. The source of such discrepancies in results is unclear, although it has been reported that subgingival microbiota could differ by geographic location, possibly due to lifestyle differences ([77], [78]).

### Potential limitation

Our relatively small sample size is largely due to our rigorous criteria for subject recruitment in China. We excluded cigarette smokers and subjects with other systemic illness. We also excluded subjects with root caries since it is well known that the pathogenic bacteria of caries are highly different from those associated with periodontitis ([79], [80]). Despite our relatively small sample size, we could still detect differences among subject groups at both the bacterial community level and the individual bacteria level. However, small sample size inevitably limits the statistical power. We might have missed some bacteria that exist at different abundances in the different sample groups.

Another limitation is the relatively short read length (i.e., around 500 bp) associated with the 454 pyrosequencing technology. The BLAST-based species-level classification of short reads can be very challenging because multiple reference sequences in the database may match equally well to the query sequence. In our study, we manually examined the BLAST alignments and classified OTUs at species level only if the annotated top hit matched better than the other database hits. Nevertheless, manual assignment is subjective by nature as in many other aspects of bioinformatics inferences where it is difficult to deploy any automatic cutoff. We have decided to provide all the OTU sequences (File S1) for other researchers to verify our species classification of their OTUs of interest and compare with the latest database records in the future.

## Conclusions

We have conducted the first high-throughput 16S rDNA pryosequencing to compare the subgingival plaque microbiota in non-diabetic subjects without periodontitis, non-diabetic subjects with periodontitis, type 2 diabetic patients without periodontitis, and type 2 diabetic patients with periodontitis. Based on the comparisons, a number of health-associated and periodontitis-associated bacteria were detected in both diabetic and non-diabetic backgrounds. In both periodontitis-negative and periodontitis-positive groups, we also detected that diabetic and non-diabetic subjects harbored bacteria at several taxonomic levels with significantly different prevalence or abundance.

## Supporting Information

Figure S1 **Distribution of all signature OTUs among four subject groups.** The bar-plot shows the distribution of all signature OTUs among the four subject groups. The bar heights correspond to relative abundance percentage, and are log-scaled. The error bar indicates one unit of standard error.
(TIF)

Table S1 **Clinical parameters of the participant subjects.** Each subject was given an ID (column 1) and assigned a group according to periodontitis and diabetes statuses. Gender and age were recorded. Probing depth (PD) indicates the average depth in millimeters of the four deepest periodontal pockets. Attachment loss (AL) indicates the average tooth support tissue loss in millimeters of the same four sites. For subjects with diabetes, fasting blood sugar (FBS), two-hour postprandial blood sugar (PBS), and glycosylated hemoglobin, the three-month average glucose level (HbA1c), were recorded.
(XLSX)

Table S2 **Classification of all signature OTUs.** These signature OTUs had significantly different abundances or prevalence in at least one of the following sample group comparisons: P−D− vs. P+D−, P−D+ vs. P+D+, P−D− vs. P−D+, or P+D− vs. P+D+ (see figure S1 for the distribution of the signature OTUs). Any significant (p<0.05) Wilcoxon rank-sum test or Fisher's exact test p-values are stated in the corresponding column. For the two comparisons of periodontal health, the “Association with periodontal health of subjects” column indicates whether the OTU is enriched in the periodontitis-negative or periodontitis-positive samples of that comparison. Similarly for the two comparisons of diabetes status, the “Association with diabetes status of subjects” column indicates the sample group with the higher abundances for that OTU.
(XLSX)

File S1 **FASTA-format sequences for all the OTUs.** Sequences for each OTU are provided in a FASTA-format file.
(ZIP)
