# Supplementary figures and images for: Investigation of the Effect of Type 2 Diabetes Mellitus on Subgingival Plaque Microbiota by High-Throughput 16S rDNA Pyrosequencing
Source: PLoS One. 2013 Apr 22;8(4):e61516. doi: 10.1371/journal.pone.0061516 (PMC3632544; doi:10.1371/journal.pone.0061516)

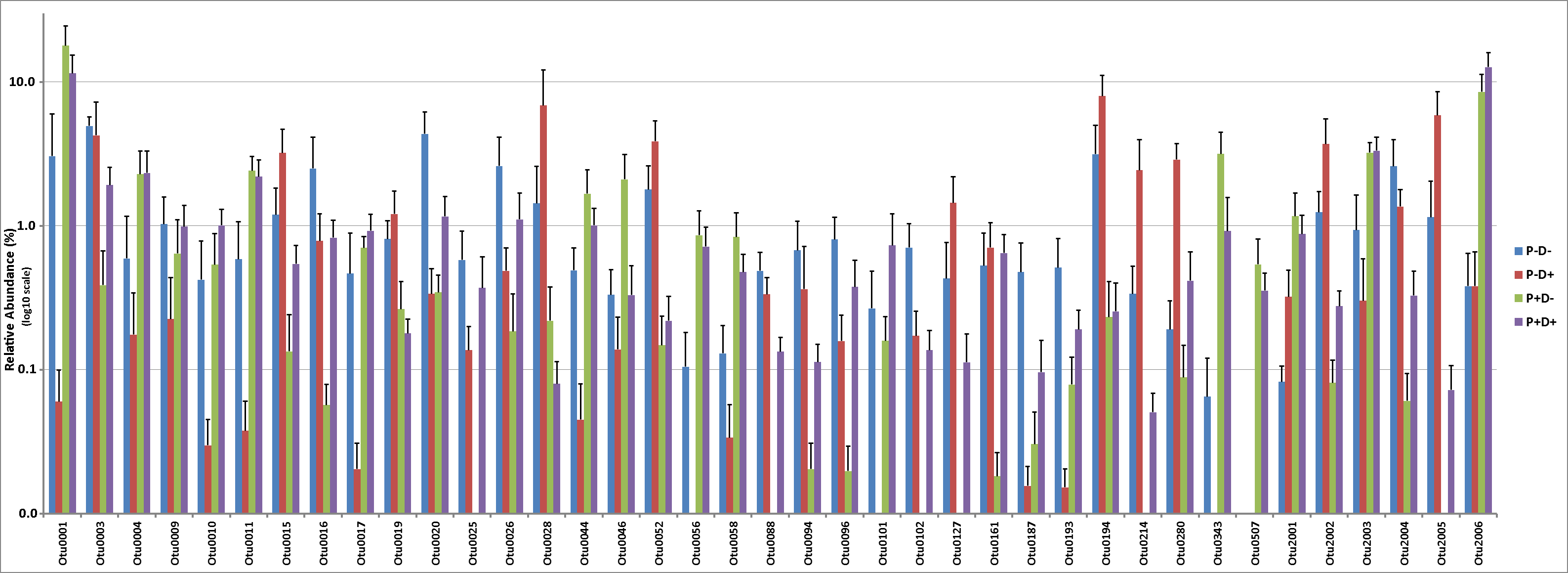

Supplement: Figure S1 — Distribution of all signature OTUs among four subject groups. The bar-plot shows the distribution of all signature OTUs among the four subject groups. The bar heights correspond to relative abundance percentage, and are log-scaled. The error bar indicates one unit of standard error. (TIF) [file pone.0061516.s001.tif]
